# Pro-Resolving Ligands Orchestrate Phagocytosis

**DOI:** 10.3389/fimmu.2021.660865

**Published:** 2021-06-10

**Authors:** Christa Decker, Sudeshna Sadhu, Gabrielle Fredman

**Affiliations:** The Department of Molecular and Cellular Physiology, Albany Medical College, Albany, NY, United States

**Keywords:** macrophage, phagocytosis, efferocytosis, resolvin, inflammation

## Abstract

The resolution of inflammation is a tissue protective program that is governed by several factors including specialized pro-resolving mediators (SPMs), proteins, gasses and nucleotides. Pro-resolving mediators activate counterregulatory programs to quell inflammation and promote tissue repair in a manner that does not compromise host defense. Phagocytes like neutrophils and macrophages play key roles in the resolution of inflammation because of their ability to remove debris, microbes and dead cells through processes including phagocytosis and efferocytosis. Emerging evidence suggests that failed resolution of inflammation and defective phagocytosis or efferocytosis underpins several prevalent human diseases. Therefore, understanding factors and mechanisms associated with enhancing these processes is a critical need. SPMs enhance phagocytosis and efferocytosis and this review will highlight mechanisms associated with their actions.

## Introduction

Elie Metchnikoff uncovered the significance of phagocytosis nearly 100 years ago (1). Since then, phagocytosis has been recognized as a critical cellular program for innate and adaptive immune responses to foreign material. Moreover, we now appreciate that engulfment and neutralization of invading organisms is key to maintain health. Phagocytes like PMN and macrophages promote microbial removal and wound debridement. Mechanisms associated with phagocytosis continue to be uncovered which has aided in our understanding of inflammation and disease. There are now numerous endogenous factors like lipid mediators (LMs), proteins, metabolites and gasses, that can promote phagocytosis. For example, LMs like specialized pro-resolving mediators (SPMs) are biosynthesized from arachidonate (AA), eicosapentaenoic acid (EPA), docosahexaenoic acid (DHA), or n-3 docosapentaenoic acid (DPA) (2, 3). SPMs are named lipoxins, resolvins, protectins, their aspirin-triggered isomers, maresins, cysteinyl-conjugated SPMs (CTRs) and 13-series resolvins (RvTs) (2, 4–9). Each of the SPMs has a distinct chemical structure (2) and several of the SPMs bind and signal through distinct G-protein coupled receptors (GPCRs) (10–14). SPMs in general exert a tissue protective action in as much as they can temper pro-inflammatory factors and promote the clearance of harmful stimuli and dead cells (i.e. phagocytosis). Pro-phagocytic ligands are not limited to SPMs and indeed the repertoire of these factors are growing and is summarized in **Table 1**.

**Table 1:** Pro-resolving factors that promote phagocytosis.

			Phagocyte	Phagocytic action elicited	References
Lipid Mediators	EPA Derived	RvE1	Macrophages (human and murine)	-Increases phagocytosis of Zymosan, E. *coli*, C. *albicans*	(15–19)
			Neutrophils (human and murine)	
				-Promotes efferocytosis of apoptotic PMN	
		RvE2	Macrophages (human)	-Increases phagocytosis of Zymosan	(20)
		RvE4	Macrophages (human)	-Increases efferocytosis of apoptotic PMN, effete RBCs	(21, 22)
	DHA Derived	RvD1, AT-RvD1	Macrophages (human, murine, rat)	- Increases phagocytosis of Zymosan, E. *coli*, P. *aeruginosa*, NTHi, IgA-OVA-coated beads, Amyloidβ, clot particles, cancer cell debris	(23–41)
			Neutrophils (human and murine)	- Promotes efferocytosis of apoptotic PMN, osteoblasts, Jurkats, macrophages, thymocytes, and sickle cell RBCs	
			Fibroblasts (human)	-Enhances clearance of necroptotic cells	
		RvD2	Macrophages (human and murine)	-Enhances phagocytosis of live *E. coli*	(29, 42–44)
			Neutrophils (human and murine)		
				-Promotes efferocytosis of apoptotic PMN apoptotic osteoblasts	
		RvD3, AT-RvD3	Macrophages (human and murine)	-Enhances phagocytosis	(40, 45)
				-Promotes efferocytosis of apoptotic PMN	
				-Increases uptake of cancer cell debris	
		RvD4	Macrophages (human)	-Enhances phagocytosis of *S. aureus, Zymosan*	(25, 46)
			Fibroblasts (Human)		
			Whole blood phagocytes		
				-Increases efferocytosis of apoptotic PMN	
		RvD5	Macrophages (human)	-Enhances phagocytosis of *E. coli*	(23)
		MaR1, MaR2	Macrophages (human and murine)	-Enhances phagocytosis of E. *coli*, Zymosan	(47–50)
				–Increases efferocytosis of apoptotic PMN	
		PD1/NPD1, AT-PD1	Macrophages (human and murine)	-Increases efferocytosis of apoptotic PMN, thymocytes	(15, 23, 41, 51–54)
		Cys SPMs:	Macrophages (human and murine)	-Enhances phagocytosis of *E. coli*, Zymosan	(8, 55–59)
		MCTR1,			
		MCTR2,			
		MCTR3,		-Increases efferocytosis of apoptotic PMN	
		PCTR1,			
		PCTR2,			
		PCTR3,			
		RCTR1,			
		RCTR2,			
		RCTR3			
	n-3 DPA derived SPMs	RvD5_n-3_DPA	Macrophages (human and murine)	-Increases phagocytosis of S. aureus, Zymosan	(9, 14, 60)
		PD1_n-3_DPA			
		Mar_n-3_DPA			
		RvD_n-3_DPA			
		RvT		-Enhances efferocytosis of apoptotic HL-60 cells	
	AA Derived	LXA_4_, AT-LXA_4_	Macrophages (human, murine, rat, THP-1 cells)	-Enhances efferocytosis of apoptotic PMN	(61–63)
		LXB_4_, AT-LXB_4_	Macrophages (human, murine, rat)	-Increases phagocytosis of E. *coli*	(63, 64)
				-Enhances efferocytosis of apoptotic PMN	
**Proteins**		Annexin A1, Ac2-26	Macrophages (human)	-Enhances efferocytosis of apoptotic PMN	(65)
		DEL-1	Macrophages (murine)	-Increases efferocytosis of apoptotic PMN	(66)
		IL-10	Macrophages (human)	-Increases phagocytosis of E. *coli*	(67)
				-Enhances efferocytosis of apoptotic PMN	
		IFN-β	Macrophages (murine)	-Enhances efferocytosis of apoptotic PMN	(68)
		Galectin-1	Microglial cells Macrophages	-Enhances phagocytosis of myelin	(69, 70)
				–Increases efferocytosis of apoptotic PMN	
		Galectin-3	Neutrophils (human)	-Enhances phagocytosis IgG-RBCs	(71, 72)
			Macrophages (murine)		
				–Increases efferocytosis of apoptotic PMN	
		Melanocortin	Macrophages (murine)	-Enhances phagocytosis of zymosan	(73)
				–Increases efferocytosis of apoptotic PMN	
		Alpha-2-macroglobin	Macrophages (murine)	-Enhances phagocytosis of zymosan	(74)
				–Increases efferocytosis of apoptotic PMN	
**Gases**		H_2_S	Macrophages	-Enhances phagocytosis of bacteria	(75)
		CO	Macrophages (human)	-Increases phagocytosis of zymosan	(76)
				–Increases efferocytosis of apoptotic PMN	
**Nucleotides**		Adenosine	PMN	-Stimulates Fc-mediated phagocytosis	(77)
**Lipids**		Estrogen	Microglial cells	-Stimulated efferocytosis of apoptotic PC12 cells	(78)

Non-resolving inflammation is an underpinning of several prevalent diseases, including cardiovascular and neurodegenerative diseases, cancer, arthritis, asthma etc. Phagocytes play a major role in the resolution of inflammation because of their ability to neutralize and contain harmful stimuli and clear dead cells and debris, which is a feed-forward process that helps repair tissue injury. Not surprisingly, phagocyte functions in several diseases mentioned above have been shown to be dysregulated and are nicely reviewed in the following reference (79). Instead, we appreciate that aging is a major risk factor for several diseases and is a scourge of modern medicine. Therefore, this mini review will highlight some of the pro-phagocytic bioactions of SPMs, with a focus on aging. Lastly, we will offer some suggestions for future studies in this arena.

## Pro-Resolving Ligands Enhance the Clearance of Microbes and Debris

We now appreciate that phagocytosis and thus the engulfment and neutralization of invading organisms is key to maintain health. There are several manners in which phagocytes like neutrophils or macrophages ingest pathogens, which is nicely reviewed by Flannagan RS et al. (80). Briefly, the removal of pathogens is a highly orchestrated event that involves cell surface receptors and specific signaling pathways to initiate recognition, engulfment and degradation. SPMs have been shown to increase phagocytosis (**Table 1**) of pathogens and some of the initial findings are described below. One of the earliest observations involved the SPM called Resolvin E1 (RvE1). RvE1 was shown to enhance the clearance of *Candida albicans* by human neutrophils and in a mouse model of candidiasis (17). In this same paper, RvE1 was also shown to be biosynthesized by C. *albicans.* Other pathogens like *Pseudomonas aeruginosa* (81), T*. gondii* (82) and T*. cruzi* (83) were also shown to biosynthesize SPMs. Collectively, these studies demonstrate an intimate link between host-derived SPMs and pathogens. Some questions remain, like why would microbes make SPMs? From the viewpoint of pathogens like Candida, RvE1 could potentially limit the number of recruited polymorphonuclear lymphocytes (PMN), allowing for its persistence. From the perspective of the host, Candida also increases IL-8 (which is a neutrophil chemoattractant) and the presence of RvE1 can enhance local phagocytes to clear Candida (17). From an evolutionary perspective, it is possible that mammalian hosts hijacked SPMs as important armament to protect against foreign invaders. Therefore it is possible that evolutionary pressures on the host are what drove SPMs to possess the ability to enhance phagocytosis. Along these lines, several SPMs such as RvD5, PD1, RvD1 and RvD2 each enhanced the clearance and neutralization of E. *coli* by neutrophils and macrophages (23, 42). An important finding was that these SPMs do not directly kill E. *coli*, but rather act on phagocytes to enhance killing in a contained manner. **Table 1** summarizes key papers associated with SPM’s ability to enhance phagocytosis in numerous contexts. SPMs also promote phagocytosis of pathogens found in the lungs (34, 84, 85), which may be important for a return to homeostasis post airway infections. Along these lines, RvD1 and Mar1 contain and limit *Mycobacterium tuberculosis* intracellular growth in macrophages (86). RvD1 increased antimicrobial peptides including bactericidal/permeability-increasing protein (BPI) and LL-37 and Mar1 only increased BPI. The control and containment of Tuberculosis relies heavily on the balance between pro-inflammatory and pro-resolving factors (87). Moreover, RvD1 and Mar1 each stimulated NF-κB nuclear translocation but only Mar1 promoted Nrf2 localization. These results suggest that while RvD1 and Mar1 have overlapping functions, they also exert distinct actions and thus highlight the utter importance of having several distinct SPM species available to promote optimal function. A deeper understanding of the common and distinct actions of SPMs on phagocytosis are of interest.

Along these lines, uncovering mechanisms associated with SPM clearance of microbes and debris is an emerging area of research. As an example, aspirin-triggered lipoxin A_4_ (or AT-LXA_4_) was shown to enhance phagocytosis in a mannose scavenger receptor dependent manner (61) because inhibition of the mannose receptor by treatment with mannin completely abrogated AT-LXA_4_’s ability to enhance the clearance of E. *coli* (61). In this same study, the authors also found that AT-LXA_4_ required PI3K and p110γ to enhance phagocytosis (61). AT-LXA_4_ binds and signals through a GPCR called ALX/FPR2 and so these results suggest that SPMs engage signaling pathways *via* their cell surface GPCRs. Accordingly, RvD2 binds and signals through a GPCR called GPR18 and knockdown of GPR18 abrogated RvD2’s ability to enhance phagocytosis (43). Moreover, RvD2 enhanced the expression of key phagocytic recognition receptors like CD206 and CD163 (43). In a subsequent study, RvD2 was also found to increase phagocytosis through mechanisms involving cAMP, PKA and STAT3 (44). Other SPMs, like 13S,14S-epoxy-DHA (or eMaR) and RvD1 also increased surface levels of CD163 and CD206 on human macrophages (88). Also, n-3 docosapentaenoic acid–derived resolvin D5 (RvD5_n-3 DPA_) enhanced phagocytosis through mechanisms involving a GPCR called GPR101 (14). Collectively, these are only a few examples of how SPMs enhance phagocytosis and further understanding of the intricate signaling pathways associated with SPM-initiated phagocytosis are of immense interest. SPMs have also been reported to enhance the clearance of debris, including fibrin clots (35), which is an important process to maintain vascular homeostasis. Overall, SPMs enhance the clearance of microbes and debris. There are several remaining questions: Do particular subsets of macrophages or neutrophils respond optimally to SPMs, and if so which species of SPM? How are SPM GPCRs regulated during phagocytosis? How do SPMs impact macrophage or PMN metabolism when ingesting and neutralizing pathogens? Addressing these questions will likely yield important information as to how cells integrate SPM signals and may help inform the development of targeted therapies for particular infections. Another critical aspect to the maintenance of homeostasis is the clearance of dead cells. The next sections will focus on how SPMs enhance dead cell removal by phagocytes.

## Pro-Resolving Ligands Enhance the Clearance of Dead Cells

Billions of cells die daily in adult lives and so the efficient clearance of dead cells is utterly critical for homeostasis (89). Moreover, a large number of cells also die during the resolution of a pathological outcome, such as infection or tissue damage (89). The engulfment of dead cells by professional phagocytes like macrophages is called efferocytosis, which is a highly intricate process that ultimately allows for the recycling of cellular products and tissue repair (79, 90). Failure to clear dead cells can lead to accumulation of necrotic debris, which is associated with several prevalent human diseases, including atherosclerosis (91). Therefore, a major topic of interest in recent years has been the exploration of factors that increase efferocytosis to quell persistent inflammation, limit tissue necrosis and promote repair.

SPMs are of immense interest because of their ability to enhance efferocytosis. Lipoxin A_4_ (LXA_4_) was among the first SPMs that was published to enhance the clearance of apoptotic cells *in vitro* (62, 92). Mechanistically, the increase in efferocytosis is in part through LXA_4_’s ability to dephosphorylate MYH9, a protein involved with cytoskeletal rearrangement (92). This dephosphorylation results in the activation of components of a signaling cascade leading to cell polarization and increased phagocytosis. In addition to MYH9, LXA_4_ also activates CDC42, which promotes engulfment (92).

Numerous SPMs, including the E-series, D-series, DPA-derived resolvins, protectins and maresins enhance the clearance of dead cells (93). This redundancy again suggests that SPMs and efferocytosis are utterly critical for tissue repair mechanisms and our survival. The mechanisms by which SPMs enhance efferocytosis are under investigation. Because SPMs have distinct structures, the first step in our understanding toward mechanisms is to investigate the receptors to which they bind. Indeed, several SPM receptors have been discovered and are highlighted in further detail by Chiang et al. (2). Removal of key receptors for SPMs demonstrate that SPMs initiate pro-efferocytotic programs through their cell surface receptors (14, 36, 43, 47).

Intracellular signaling associated with SPM mechanisms are also of immense interest. Resolvin D1 for example binds and signals through a GPCR called ALX/FPR2 (36). RvD1 initiates a cAMP-PKA signaling event and also limits the phosphorylation of p47, a crucial molecule involved in NADPH oxidase (NOX) activation (37). Limiting the activation of NOX aids in quelling inflammation by reducing the amount of ROS which trigger oxidative stress induced cellular damage (37). To this end, it is appreciated that certain pro-inflammatory cytokines, like TNF-α can limit efferocytosis through increased ROS in macrophages (94). RvD1 also limits LPS-induced TNF-α expression to rescue defective efferocytosis by controlling the classical NF-_k_B1 pathway and activating an atypical pathway that suppresses the secretion of TNF-α and IL-1β (28). Moreover, through C-terminal cleavage of NF-_k_B1, RvD1 initiates formation of a p50/p50 homodimer which competes for DNA binding with the classical heterodimer (28). Therefore, RvD1’s actions in limiting the release of pro-inflammatory factors (28, 37, 95–97) may also aid in its ability to enhance efferocytosis. Another interesting angle is that oral administration of RvD1 was recently shown to control key transcriptional profiles in ingesting macrophages *in vivo* (98). For example, RvD1 reduced transcript levels of coactivator‐associated arginine methyltransferase 1 (CARM1), histone aminotransferase 1 (HAT1), histone deacetylase 5 and 7 (HDAC5 and HDAC7), protein arginine *N*‐methyltransferase 2 (PMRT2), and ribosomal protein S6 kinase, polypeptide 5 (RPS6KA5) in macrophages from inflammatory loci *ex vivo* (98). With regard to the regulation of CARM1, adoptive transfer of siCARM1‐transfected human macrophages to zymosan‐challenged mice resulted in a significant increase in PMN clearance, which suggests an important counter regulatory role for CARM1 and efferocytosis (98). RvD1 also decreased HDAC5 and HDAC7, which suggests that RvD1 may regulate epigenetic mechanisms (98). How RvD1-initated epigenetic control of macrophages impacts its efferocytic function is of interest. Along these lines, RvD1 also regulated a panel of miRNAs that might contribute to phagocytosis, efferocytosis and resolution of inflammation (99) and a deeper exploration of roles and mechanisms of pro-resolving and pro-phagocytic miRNAs are also of interest.

Another emerging area of interest is the macrophage’s ability to carry out continual efferocytosis, which is the ability of individual macrophages to engulf multiple apoptotic cells consecutively. Early work in this arena demonstrated that CD11b levels were associated with a macrophage’s ability to eat “a little” or “a lot” of apoptotic cells (100). We now appreciate that macrophages within tissues are very diverse with regard to function and phenotype and newer research suggests that macrophage metabolism is an important player in the ability of a macrophage to eat apoptotic cells (101–105). Apoptotic cell engulfment and the eventual breakdown of the corpse provides the fuel for metabolic programs like fatty acid oxidation (FAO) and oxidative phosphorylation (OXPHOS) (101). Macrophages also engage aerobic glycolysis to ingest apoptotic cells (105). Recent work by Yurdagul A et al. demonstrates a critical role for amino acid metabolism and continual efferocytosis (102, 103). Briefly, they found that arginine can be metabolized to ornithine by Arginase-1 in pro-resolving macrophages. Moreover they also found that pro-resolving macrophages converted ornithine into putrescine *via* ornithine decarboxylase (ODC) for continual efferocytosis. To further this mechanism they then found that ODC-dependent putrescine synthesis drives IL-10 production and inflammation-resolution *in vivo*. Moreover, putrescine promoted MerTK levels through sH3K9 di/trimethylation mechanisms. Together, these results uncover key players in our understanding of the link between macrophage metabolism, efferocytosis and inflammation resolution programs.

## Efferocytosis Promotes SPM Biosynthesis

Another fascinating finding is that the process of efferocytosis itself leads to the biosynthesis of more SPMs (15, 106, 107). This feed-forward circuit was first demonstrated in murine systems (15). Human macrophages that had ingested apoptotic PMN also had increased biosynthesis of SPMs, including RvD1, RvD2 and LXB_4_ (106). A potential mechanism for the increase in SPMs during efferocytosis is transcellular biosynthesis (106). From a mechanistic perspective and to determine whether released SPMs enhance efferocytosis in a feed forward manner, Chiang N et al. knocked down a key SPM biosynthetic enzyme called 15-lipoxygenase (15-LOX) in human macrophages. They found the 15-LOX silenced macrophages had significantly impaired efferocytosis, which suggests a critical role for SPM synthesis and efficient clearance (76). Another mechanism that promotes SPMs during efferocytosis is through MerTK signaling (107). Cai B. et al. demonstrated that MerTK signaling led to increased SPMs and that silencing of MerTK resulted in less SPMs (107). Mechanisms through which MerTK increases SPMs may be through non-nuclear subcellular localization of a key SPM biosynthetic enzyme called 5-lipoxyeganse (5-LOX) (95, 107). Collectively, these papers point to a critical feed-forward mechanism in which efferocytosis stimulates SPMs and SPMs act locally to further enhance efferocytosis.

## Released SPMs as Important“Good-Bye” Tissue Messengers

SPMs and other lipid mediators were also shown to be released by apoptotic neutrophils (106). Interestingly some SPMs enhance the migration of monocytes and macrophages and so released SPMs by apoptotic cells may act as a signal for phagocytes to find these cells for swift clearance. Moreover, these findings suggest that apoptotic cells themselves are active participants in their own clearance. Newer work suggests that metabolites such as putrescine released by apoptotic cells also participate in their own clearance (108) and so released products from apoptotic cells may play critical roles in their swift clearance. More work regarding apoptotic cell secretomes and how released factors impact their clearance are of interest. In fact, Medina et al. profiled the metabolite secretome of apoptotic lymphocytes and macrophages and showed specific metabolites released by apoptotic cells act as “good-bye” messengers to modulate tissue functions. These metabolites reprogram the genes of neighboring healthy cells to facilitate an anti-inflammatory phenotype and promote tissue homeostasis (108).

Moreover, we know there are many modes of cell death beyond apoptosis. Lytic cell death like necroptosis, impacts phagocyte function given the pro-inflammatory nature of their death (109). In addition to the release of DAMPs, cytokines and chemokines, recent work from our lab suggests that necroptotic cells release prostanoids (26). Advanced atherosclerotic plaques from hypercholesterolemic *Mlkl^-/-^* mice had significantly more prostanoids like PGE_2_, PGD_2_, PGF_2α_, and thromboxane (TX) compared with wild type (Wt) controls (26). In vitro studies revealed that necroptotic macrophages and endothelial cells also released prostanoids, including TX and PGE_2_ (26). Receptor antagonists for DP (i.e. PGD_2_ receptor), EP2 (i.e. PGE_2_ receptor) and TP (i.e. TX receptor) rescued defective efferocytosis caused by necroptotic cell releasate, suggesting that a prostanoid storm negatively impacts phagocyte behavior (26). Moreover, macrophages stimulated with a TP agonist called U46619 impaired the clearance of both apoptotic and necroptotic cells and so TX may be a novel “avoid me” signal (26). As mentioned above, certain pro-inflammatory cytokines also limit efferocytosis and so released factors from necroptotic cells (or cells that undergo a pro-inflammatory mode of death) may play a large role in negatively impacting phagocyte behavior in tissues.

## SPMs Promote the Clearance of Necroptotic Cells

Necroptosis is a pro-inflammatory form of cell death (110, 111) and so the accumulation of necroptotic cells can be damaging to tissues (112). Therefore, uncovering mechanisms associated with necroptotic cell clearance is of immense interest. Earlier work demonstrated that necroptotic cells were cleared by macrophages in a distinct and less efficient manner than apoptotic cells (113, 114). However, detailed molecular mechanisms, quantification methods, and factors that augment the clearance of necroptotic cells were not known. We recently found that necroptotic macrophages express high levels of a “don’t eat me” signal called CD47 (27). Other work suggests that the exuberant expression of CD47 results from pro-inflammatory cytokines, like TNF-α (115), and so the pro-inflammatory nature of this cell death likely drives the increase of CD47. Moreover, because necroptotic cells have regions of disrupted membrane (109), we also found that elevated levels of CD47 were present in clusters on the surface of the necroptotic cell (27). The elevated levels and clustering of CD47 led to an inefficient “nibbling” of necroptotic cells *via* a RhoA-pMLC signaling event (27). RvD1 when given *in vitro* and *in vivo* was able to enhance the clearance of necroptotic cells by promoting whole cell engulfment (26, 27). RvD1-stimulated whole cell engulfment of necroptotic cells by macrophages was non-phlogistic and RvD1 acted by limiting RhoA-pMLC signaling and promoting CDC42 (27). Additionally, RvD1-stimulated macrophages swiftly recognized necroptotic cells for their engulfment and how RvD1 overcame the “don’t eat me” recognition was of interest. ER-mediated phagocytosis is a process in which macrophages release calreticulin onto their target. In other contexts, ER-mediated phagocytosis has been described and is thought to be important for eating large cargo (116, 117). Our work suggests that RvD1 promotes the release of calreticulin from macrophages (117) and may be a mechanism through which RvD1 can swiftly recognize these cells for whole-cell clearance. This work highlights the intricate set of signals/signaling that a macrophage needs to decode for efficient engulfment and clearance of dead cells.

From a metabolic perspective RvD1 stimulates p-AMPK, fatty acid oxidation (FAO) and oxidative phosphorylation (OXHPOS) mechanisms in macrophages to allow for enhanced clearance of necroptotic cells (26). We found OXPHOS is not as readily activated in vehicle-treated macrophages that were ingesting necroptotic cells (26), which suggests that the cargo load within the macrophage may be important for initiating these programs. As mentioned above, apoptotic cell uptake and its eventual breakdown provides the fuel for FAO and OXPHOS in macrophages (101) and so these data suggest that RvD1 maintains this protective metabolic phenotype.

## Pro-Resolving Ligands Rescue Age-Related Defects in Efferocytosis

The population is rapidly aging, health care costs are already insurmountable and therapeutics to manage several age-related diseases are limited. Therefore aging and age-related diseases are the scourges of modern medicine. Aging is a complex process that involves genetic, environmental and biological factors. Therefore understanding a common mechanism that may link all of these diseases will inform the development of therapeutics that can promote health span. Persistent, non-resolving inflammation, or inflammaging, largely contributes to a panoply of age-related diseases including periodontal disease, neurodegenerative diseases, macular degeneration, and atherosclerotic cardiovascular disease (118). Defective efferocytosis in aging is well appreciated (119–123). Linehan et al. observed that peritoneal macrophages from old mice had diminished efferocytosis compared with peritoneal macrophages from young mice (121). Interestingly, they transferred peritoneal macrophages from young mice into the peritoneum of old mice, and found that the young macrophages exhibited defective efferocytosis similar to that of macrophages from old mice (121), which suggests that the aging milieu drives defective efferocytosis (120, 121). Lung macrophages from old mice also exhibited defective efferocytosis, which may account for the accumulation of leukocytes and failed tissue repair in lungs from influenza-infected aged mice (124, 125).

Accumulation of senescent cells is also associated with inflammaging (126). Senescent cells acquire a senescence-associated secretory phenotype (SASP) which exacerbates inflammation (126) and contributes to inefficient clearance of dead cells (38). Briefly, we found that released factors from senescent cells promote the cleavage of a critical efferocytosis receptor on macrophages called MerTK to limit efferocytosis (38). RvD1 rescued senescent cell-induced defective efferocytosis *in vitro*. RvD1 treatment to old mice also increased *in situ* efferocytosis in lungs post hind-limb ischemia-reperfusion injury (38). MerTK is an interesting efferocytosis receptor because of its ability to stimulate a pro-resolution feed-forward circuit. In this regard, MerTK cleavage is associated with delayed temporal resolution, impaired SPM synthesis over pro-inflammatory lipid mediators and has been shown to promote atherosclerosis (107, 127). Also, with regard to other TAM receptors and aging, Frisch et al. found that Gas6, which is a ligand for MerTK (and other TAM receptors), was down regulated in the bone marrow from aged mice (123) which may account for defective efferocytosis in the aging bone marrow milieu. Moreover, they demonstrated loss-of-function of another efferocytic TAM receptor, Axl in bone marrow macrophages from aged mice which further leads to a significant increase in pro-inflammatory IL-1β signaling. Together, these results suggest that efferocytosis receptors and thus a feed-forward pro-resolution circuit may be dysfunctional in aging.

**Figure 1:**
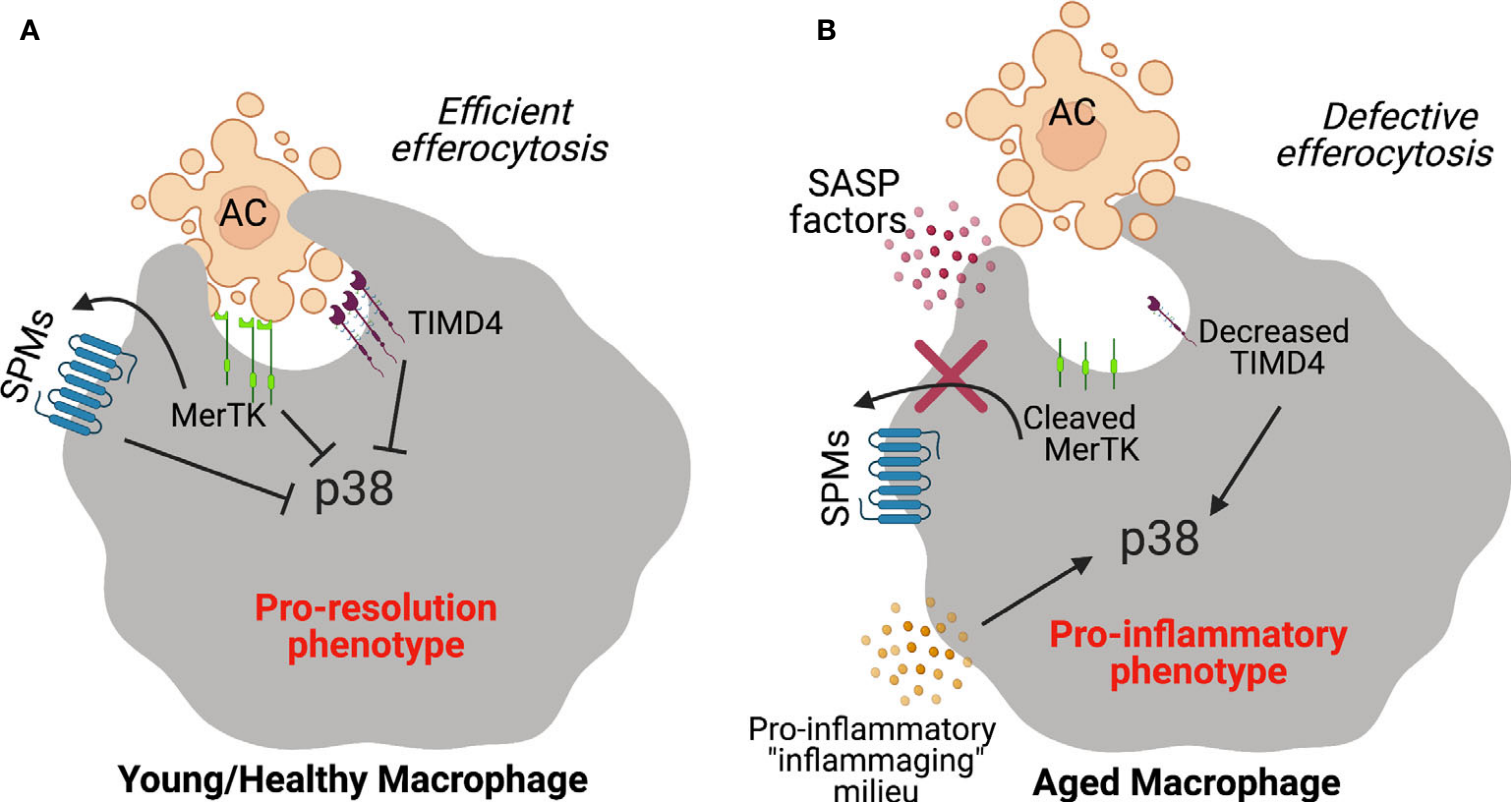
Schematic diagram depicting failed efferocytosis mechanisms during aging. **(A)** Efferocytic receptors like MerTK or TIMD4 on young healthy macrophages interact with phosphatidyl serine on apoptotic cells to promote efficient clearance. SPMs promote efferocytosis and MerTK signaling in a feed forward manner which stimulates the synthesis of SPMs. In response to apoptotic cell ingestion, the pro-inflammatory p38 MAPK pathway is inhibited and macrophages prevent the production of pro-inflammatory programs. **(B)** In the context of aging, MerTK is cleaved by released factors from senescent cells (i.e. factors from the senescence-associated secretory phenotype or SASP) which limits apoptotic cell uptake and the feed-forward pro-resolution circuit. TIMD4 expression is also significantly decreased on macrophages from aging humans which drives the activation of p38 to propagate inflammation.

Along these lines, imbalances in the SPM to pro-inflammatory lipid mediator ratios have been observed in mice and humans in the context of inflammaging (122, 128). In elderly humans, urinary lipoxins (LXs) were decreased resulting in a profound imbalance between pro-resolving LXs and leukotrienes (LTs) (128). Other relevant age-related diseases like atherosclerosis, peripheral vascular disease, periodontal disease and Alzheimer’s disease are also associated with imbalances in this critical ratio and restoration of defective SPMs to these pre-clinical models of disease result in protection (129–132). Nevertheless, how these imbalances arise in aging is of interest but work suggests that one mechanism (of many) may be through MerTK signaling (38). MerTK signaling in human macrophages decreased activity of the mitogen-activated protein kinase (MAPK) p38 and the kinase MK2, resulting in the increased non-phosphorylated, cytoplasmic form of 5-LOX and enhanced SPM biosynthesis (133). Therefore, downstream signaling events from efferocytic responses may drive SPM synthesis of pro-inflammatory mediators. Of note, RvD1 was shown to limit p38 activation which promotes a pro-resolution circuit in macrophages (95). Therefore, continued activation of p38 may not only drive defective efferocytosis but also impair resolution in aging (**Figure 1**).

Recent work demonstrated that the efferocytosis receptor, TIM-4 was decreased on macrophages from elderly humans in self-limited inflammatory loci (134). Reduced levels of TIM4 in elderly humans was attributed to increased p38 activation (134) and blockade of elevated p38 restored efferocytosis in the elderly. This work provides strong rationale for therapeutic strategies that target p38 to promote efferocytosis in aging (135). Together, phagocyte function in aging is impaired, which may be one of several factors that contributes to limited tissue repair in aging. A deeper exploration of efferocytosis mechanisms in aging may help inform the development of new tissue-reparative therapies.

## Closing Remarks: Therapeutic Opportunity

SPMs and other pro-resolving ligands offer tremendous opportunities for therapeutic use. Currently, treating inflammation is difficult because we evolved inflammatory reactions to fight infection and repair wounds. Therefore anti-inflammatories possess a risk in which critical host defense mechanisms are weakened. Anti-inflammatories may halt the progression of ongoing inflammation, but do very little to repair the already damaged tissue. Ultimately what is lacking is a therapeutic strategy that can repair tissue damage once it has occurred. SPMs are protective in numerous pre-clinical models of disease and most recently have been shown to reduce PMN infiltration during acute sterile inflammation in humans (136). SPMs stimulate phagocytes to clear, and neutralize pathogens or accelerate the removal of unwanted dead cells or debris, all while dampening inflammatory mediators and stimulating necessary pro-repair factors (58). Thus, SPMs offer an entirely new way to control inflammation which includes enhancing the removal of harmful stimuli and promoting tissue repair.

## Author Contributions

CD, SS and GF wrote the manuscript. All authors contributed to the article and approved the submitted version.

## Funding

This work was supported by NIH grants HL141127 (GF), HL153019 (GF).

## Conflict of Interest

The authors declare that the research was conducted in the absence of any commercial or financial relationships that could be construed as a potential conflict of interest.
